# Cannabinoid-induced increase of quantal size and enhanced neuromuscular transmission

**DOI:** 10.1038/s41598-018-22888-4

**Published:** 2018-03-16

**Authors:** Marco Morsch, Dario A. Protti, Delfine Cheng, Filip Braet, Roger S. Chung, Stephen W. Reddel, William D. Phillips

**Affiliations:** 1 Department of Biomedical Sciences, Faculty of Medicine & Health Sciences, Macquarie University, Sydney, NSW 2109 Australia; 2 Discipline of Physiology and Bosch Institute, The University of Sydney, Sydney, NSW 2006 Australia; 3 School of Medical Sciences (Discipline of Anatomy and Histology), The Bosch Institute, The University of Sydney, Sydney, NSW 2006 Australia; 4 Australian Centre for Microscopy & Microanalysis (ACMM), The University of Sydney, Sydney, NSW 2006 Australia; 5 Departments of Molecular Medicine & Neurology, Concord Clinical School, The University of Sydney, Sydney, NSW 2006 Australia

## Abstract

Cannabinoids exert dynamic control over many physiological processes including memory formation, cognition and pain perception. In the central nervous system endocannabinoids mediate negative feedback of quantal transmitter release following postsynaptic depolarization. The influence of cannabinoids in the peripheral nervous system is less clear and might have broad implications for the therapeutic application of cannabinoids. We report a novel cannabinoid effect upon the mouse neuromuscular synapse: acutely increasing synaptic vesicle volume and raising the quantal amplitudes. In a mouse model of myasthenia gravis the cannabinoid receptor agonist WIN 55,212 reversed fatiguing failure of neuromuscular transmission, suggesting future therapeutic potential. Our data suggest an endogenous pathway by which cannabinoids might help to regulate transmitter release at the neuromuscular junction.

## Introduction

Cannabinoids are ubiquitous regulators of synaptic transmission in the brain: acutely modulating neurotransmitter release, mediating numerous forms of short- and long-term plasticity, and strongly influencing synapse formation and neurogenesis^1,2^. While cannabinoids can act directly upon some ion channels, in most cases they modulate synaptic transmission via G-protein-coupled cannabinoid receptors^3,4^. The endogenous cannabinoid (endocannabinoid) system plays a well-established role as a retrograde regulator of synaptic plasticity in the brain^5^. Endocannabinoids are produced on demand following postsynaptic depolarization and generally act via presynaptic cannabinoid-1 (CB1) receptors to acutely increase K^+^ currents and inhibit Ca^2+^ currents^6–8^, and consequently reduce the number of quanta released during neurotransmission^1^.

While cannabinoids have been intensely studied in the brain for many years, their effects on the mammalian neuromuscular junction (NMJ) have been curiously overlooked. The NMJ provides the essential link between motor nerves and skeletal muscle and has been central to our understanding of fast communication throughout the nervous system. Cannabinoids such as 2-Arachidonoylglycerol (2-AG) and WIN 55,212 (WIN) act on central synapses by reducing the amount of transmitter released per nerve impulse (quantal content). Studies of the NMJs of lower vertebrates similarly revealed a reduction of the quantal content (QC) upon application of cannabinoids such as 2-Arachidonoylglycerol and WIN^9,10^. Sanchez-Pastor *et al*. reported reductions in both frequency and amplitude of spontaneous miniature endplate potentials (mEPPs) at the frog NMJ^11^. In contrast, a 1970’s study on the rat NMJ reported that delta9-tetrahydrocannabinol (THC), the major psychoactive component of marijuana (*Cannabis sativa*)^12^, elevated the amplitude of the spontaneous miniature endplate potential (mEPP) without altering the evoked EPP^13^. Little else is known of the actions of cannabinoids at the mammalian NMJ.

CB1 receptors are amongst the most abundant G-protein coupled receptors in the CNS^14^, being expressed at high levels by neurons in the hippocampus, cerebellar cortex, amygdala, striatum, hypothalamus, cerebellum and the dorsal horn of the spinal cord and at lower levels in the peripheral nervous system^15^. CB1 receptors are found to control the release of GABA and glutamate as well as neuromodulators such as serotonin, acetylcholine, dopamine, opioids, norepinephrine, and cholecystokinin^15,16^. CB2 receptors were initially identified on immune cells^17^. CB2 receptors are also detectable on neurons and glia in the CNS (particularly in the cerebellum and brainstem) but at much lower levels compared to CB1 receptors^18,19^. Given the well-established role of cannabinoids in negative-feedback regulation of transmitter release at central synapses we set out to investigate their function at the mouse NMJ.

Here we show that cannabinoids have a different effect at the mammalian NMJ compared to synapses in the brain. Cannabinoids increased the miniature end plate potential, which represents the quantal amplitude, and the evoked end plate potential, thereby enhancing neuromuscular transmission. We show that the increase in quantal amplitudes could be explained by an increase in synaptic vesicle volume. In a mouse model of myasthenia gravis, acute cannabinoid treatment restored disease-impaired neuromuscular transmission. We therefore conclude that cannabinoids positively modulate synaptic transmission at the mammalian NMJ through a hitherto undescribed mechanism. Our results suggest that cannabinoids might play a role in sustaining neuromuscular transmission.

## Results

### Cannabinoids elevate endplate potential amplitudes at the neuromuscular junction

**Figure 1:**
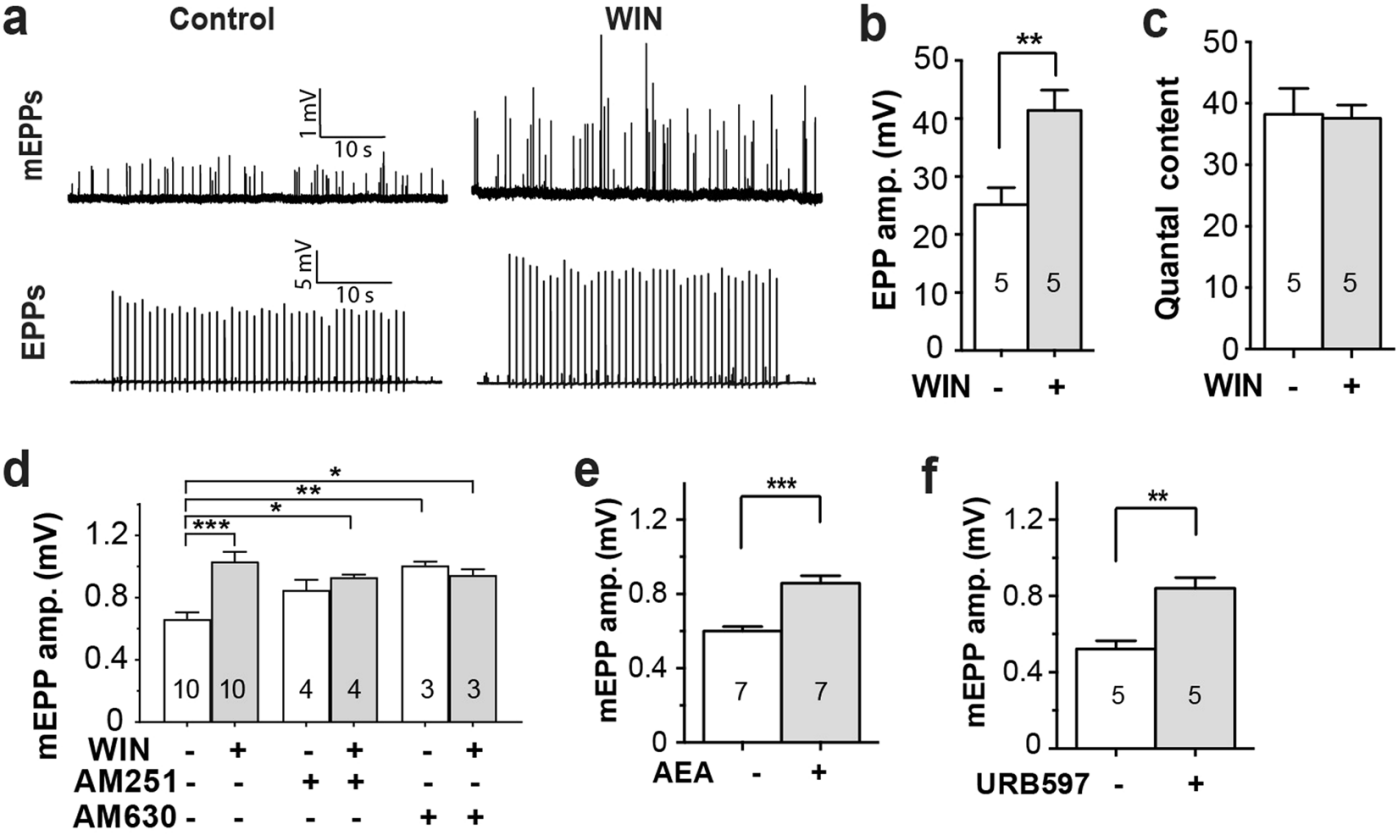
Cannabinoid-induced increase in endplate potential amplitudes. **(a**) Representative recordings show the increase in mEPP and EPP amplitudes after 1–2 hr incubation with 10 µM WIN. (**b**) WIN increased mean EPP amplitude. (**c**) No change in quantal content. (**d**) WIN increased mEPP amplitude but this effect was ablated in the presence of inverse agonists of CB1 (5 µM AM251) or CB2 (10 µM AM630). (**e**) The endogenous cannabinoid, AEA (30 µM) also increased mEPP amplitude. (**f**) An FAAH inhibitor (1 µM URB59), similarly increased mEPP amplitude. Bars represent the mean ± SEM for the number of mice indicated in each bar (average of 10–17 fibres/NMJs for each mouse/muscle; *P < 0.05, **P < 0.01; ***P < 0.001; paired t-test (**b**,**c e** & **f**), or one-way ANOVA (**d**)).

We investigated the effects of cannabinoids upon the mouse NMJ. The synthetic cannabinoid receptor agonist WIN 55,212 (WIN; 10 μM) produced 1.6 fold increases in the amplitudes of both the evoked EPPs (P < 0.01; Fig. 1a,b) and spontaneous mEPP (P < 0.001; Fig. 1a,d) approximately 1–2 hours after the addition of WIN to the *ex vivo* preparation. Unlike for many central synapses, WIN produced no change in quantal content (Fig. 1c). The increase in EPP amplitude could be fully explained by a matching increase in mEPP amplitude (P < 0.001; Fig. 1a,d). Cannabinoids can act directly upon some ion channels, but in most cases they act via the G-protein-coupled cannabinoid receptors (CB1 or CB2)^20,21^. We tested the effect by adding inverse agonists of the CB1 receptor, AM251, and the CB2 receptor, AM630, to the bath solution. The CB2 inverse agonist AM630 (10 µM) produced a significant rise in mEPP amplitude. The pharmacology of the cannabinoid receptors is complex and at this widely used concentration AM630 might act as a CB1 agonist^22^. In any event, WIN produced no significant additional increase in the mEPP amplitude in the presence of either AM251 (5 µM) or AM630 (10 µM) (Fig. 1d). Neither WIN, AM251 nor AM630 caused any change in resting membrane potential, mEPP frequency, or rise- or fall-times, (Supplementary Table 2). The endogenous cannabinoid, anandamide (AEA; 30 µM) raised mEPP amplitudes 1.4-fold, similar to the effect of WIN (Fig. 1e). Next we used a specific inhibitor of fatty-acid amide hydrolase (FAAH), URB597, to block degradation of endogenous AEA within the muscle^23^. Bath application of URB597 (1 µM) led to a 1.6-fold rise in mEPP amplitude, mimicking the effects of exogenous AEA (Fig. 1f). Together, these results demonstrate that WIN can enhance quantal amplitude at the NMJ and that endogenous cannabinoids such as AEA are generated in close proximity to act in the same way.

### The cannabinoid WIN increases presynaptic vesicle size and transmitter release

**Figure 2:**
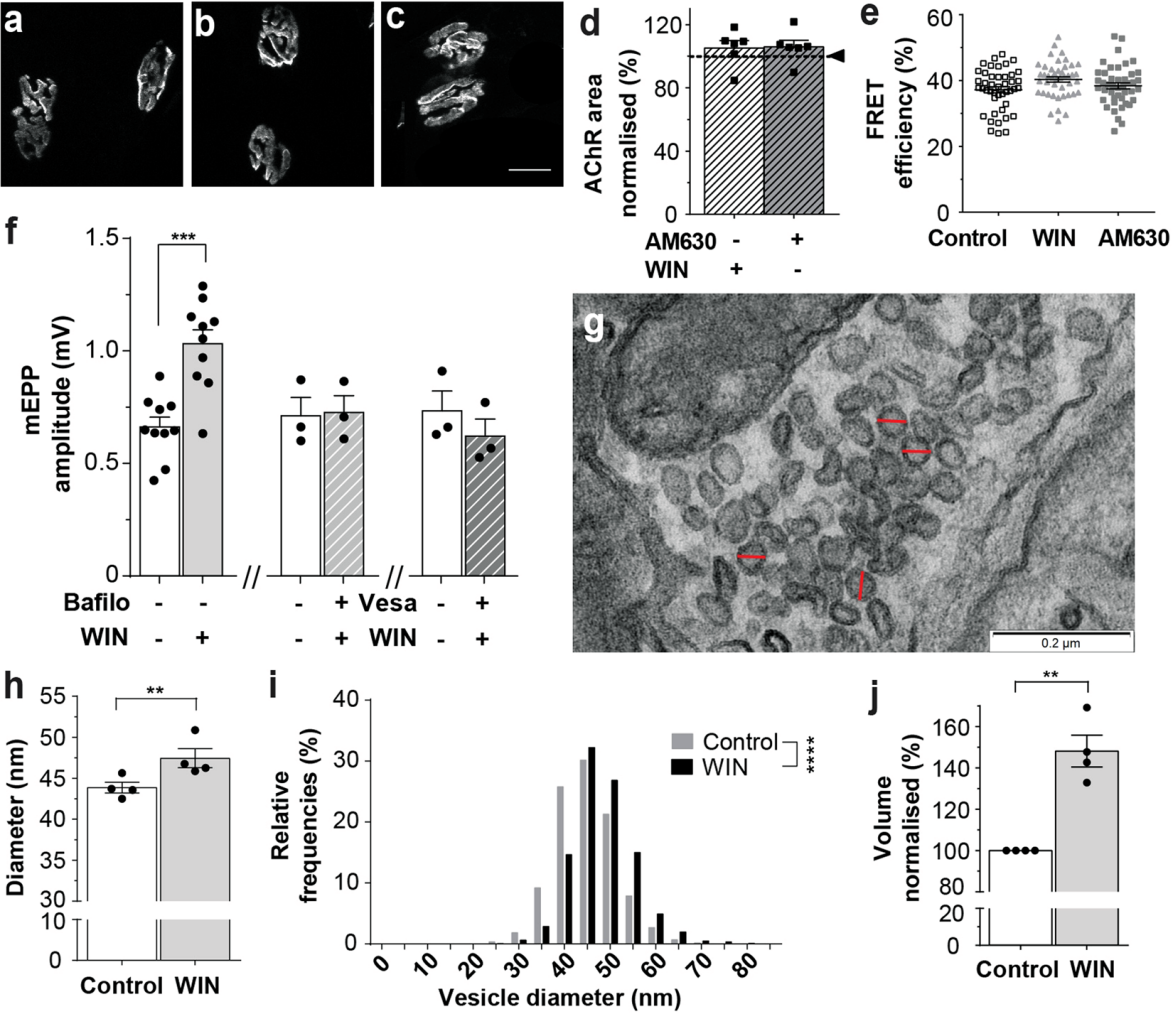
WIN acts presynaptically to increase synaptic vesicle size. (**a**–**c**) Motor endplates labeled for AChR before (**a**), or after 1.5 hrs exposure to 10 µM WIN (**b**), or 10 µM AM630 (**c**; Scale bar = 10 µm). (**d**) Postsynaptic AChR area (horizontal line shows mean for untreated controls). (**e**) Confocal fluorescence resonance energy transfer (FRET) revealed no change in AChR packing density (symbols represent individual NMJs). (**f**) The WIN-induced increase in mEPP amplitude was blocked in the presence of either bafilomycin (Bafilo; 0.1 µM) or vesamicol (Vesa; 4 µM; symbols show means for individual mice). The pair of bars at left reproduce results from Fig. 1d. (**g**) Representative transmission electron micrograph used for synaptic vesicles measurements. Red lines in panel g illustrate measurements taken from representative circular vesicles used for analyses. (**h**) Mean outer vesicle diameter before and after WIN treatment (10 µM, ~1.5 hrs; n = 4 diaphragm preparations). (**i**) Frequency distribution of vesicle diameters. Note the shift to larger diameters after WIN exposure. (**j**) Increase in calculated vesicle volume after WIN treatment. Bars represent the mean ± SEM for the number of mice indicated by each dot (mEPPs were sampled from n = 10–17 fibers for each muscle in panel f; **P < 0.01; ***P < 0.001; paired t-test (f, h & j), or unpaired t-test (i)).

WIN (10 µM) and AM630 (10 µM) caused no change in the area or density of postsynaptic nicotinic acetylcholine receptors (AChR) that might explain the increase in mEPP amplitude (Fig. 2a–e; Supplementary Fig. S1). WIN also did not enhance mEPP amplitudes by prolonging the actions of acetylcholine in the synaptic cleft (Supplementary Fig. S2), a well-described effect of cholinesterase inhibitors. By exclusion, these results suggest that WIN acts presynaptically to increase quantal size. Since the amount of acetylcholine released from each synaptic vesicle cannot be measured directly, we instead tested the effect of drugs that block the filling of synaptic vesicles. Vesamicol (4 µM) inhibits the vesicular acetylcholine transporter, while bafilomycin (0.1 µM) blocks the H^+^-ATPase that is needed to drive the acetylcholine transporter^24,25^. Neither of these drugs on their own affected mEPP amplitudes in our recordings (Supplementary Fig. S3a and see refs^24,25^), but both inhibitors blocked the WIN-induced increase in mEPP and EPP amplitudes (Fig. 2f, Supplementary Fig. S3). Thus, the cannabinoid-induced increase in quantal amplitude appears to depend upon the active transport of acetylcholine into synaptic vesicles. Consistent with enhanced vesicle filling, electron microscopy revealed an increase in synaptic vesicle diameter after WIN treatment^26^ (Fig. 2g–i; 43.9 nm vs 47.4 nm; P < 0.01). Assuming spherical geometry, this equates to a 48% increase in vesicle volume (Fig. 2j; see Methods and Supplementary Table 1). Together, our results strongly suggest that WIN acutely enhances neuromuscular transmission by increasing the loading of synaptic vesicles within the presynaptic nerve terminal.

### Impaired neuromuscular transmission in myasthenic mice is reversed by cannabinoid treatment

**Figure 3:**
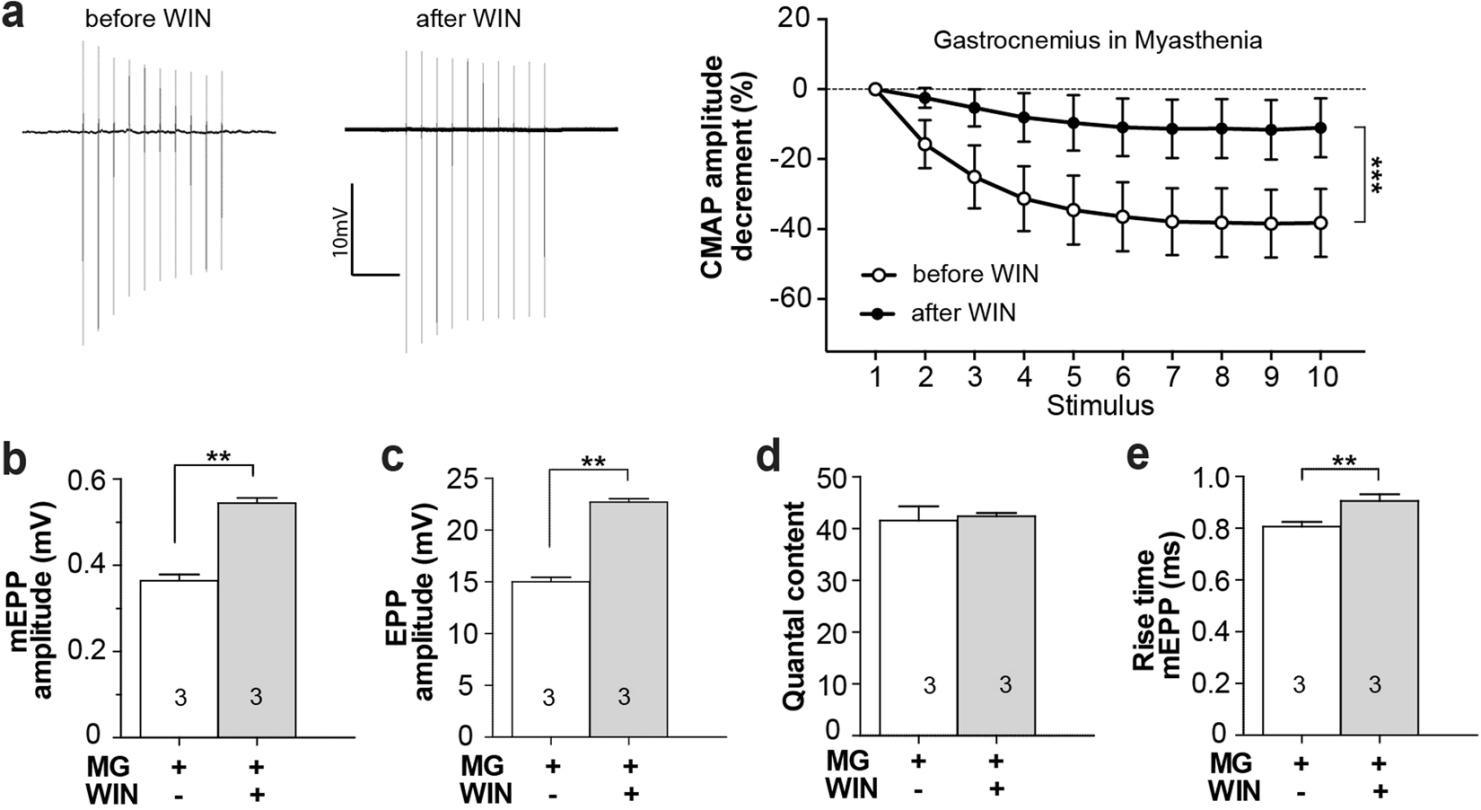
WIN reverses synaptic impairment in a mouse model of Myasthenia gravis. (**a**) Decrement in the amplitude of compound muscle action potential (CMAP) recorded from the gastrocnemius muscle of a myasthenic mouse during repetitive stimulation of the sciatic nerve. Left: Representative CMAP recordings during repetitive stimulation (3/sec) from the same myasthenic mouse before (left) and after (right) i.p. injection of WIN (5 mg/kg). Win-treated mice showed overall less fatigue of neuromuscular transmission 3 hrs after intraperitoneal injection of WIN (compare filled circles to open circles). (n = 4 mice; paired t-test of plateau amplitude; P < 0.001). (**b**–**e**) *Ex vivo* WIN treatment (10 µM, 1.5 hrs) of diaphragm muscles from myasthenic mice caused acute increases in: mEPP amplitudes (**b**), EPP amplitudes (**c**), and mEPP rise time (**e**), but not quantal content (**d**; **P < 0.01; paired t-test). Bars represent the mean ± SEM for the number of mice indicated in each bar (n = 12–16 fibers for each mouse).

In myasthenia gravis (MG) autoantibodies deplete postsynaptic nicotinic acetylcholine receptors (AChRs), thereby reducing quantal amplitude^27^. We tested the potential of WIN in a mouse model of myasthenia gravis. Mice received daily injections of IgG from an anti-MuSK-positive MG patient. The autoantibody-induced failure of neuromuscular transmission was demonstrated by a decrement in the compound muscle action potential (CMAP) during repetitive stimulation^28^ (Fig. 3a left trace and open circles). However, three hours after receiving an intraperitoneal injection of WIN (5 mg/kg), the CMAP decrement was significantly ameliorated compared to recordings from the same myasthenic mouse prior to treatment (Fig. 3a; 27% ± 8% increase in plateau amplitude; P < 0.001, n = 4 mice; paired t-test). After CMAP assessment diaphragm muscles of these myasthenic mice were dissected and tested again for responsiveness to cannabinoid stimulation. The diaphragm preparations acutely responded to reapplication of WIN (10 µM ≥1.5 hrs) with a 1.5 fold increase in the amplitudes of both mEPPs and EPPs, consistent with the effects of WIN in healthy muscles (Fig. 3b–d). In these myasthenic muscles WIN also produced a small (12%) increase in mEPP rise-time (Fig. 3e). These results demonstrate the potential for cannabinoids to ameliorate failing neuromuscular transmission in a mouse model of MG.

## Discussion

Here we report a hitherto unknown synaptic effect whereby cannabinoids acutely elevated quantal amplitudes via a mechanism that changed the filling of synaptic vesicles and lead to an expansion of vesicle volume. These effects of WIN and AEA at the mammalian NMJ differ from the classical modulatory effect of cannabinoids at central synapses, where cannabinoids generally act via presynaptic CB1 receptors to acutely reduce the number of quanta released^1^.

The effects of WIN on synaptic potentials were recorded approximately 1–2 hours after the addition of WIN to the *ex vivo* phrenic nerve-diaphragm muscle preparation. This delay was intended to ensure complete diffusion of the bath-applied WIN into the muscle such that a steady state would exist during the extended timespan of recordings from 12–16 muscle fibers. We attempted to determine the kinetics of the WIN effect by recording mEPPs from single impaled muscle fibers for 15–60 mins immediately after application of WIN. The few fibers that yielded a steady resting potential over this time frame revealed no immediate effect of WIN on mEPP amplitude or frequency. These results suggest that some time is required for the increase in vesicle filling following exposure to WIN.

Inhibition of the AEA-degrading enzyme FAAH likewise elevated mEPP amplitude, suggesting that endogenous cannabinoids can regulate quantal size at the NMJ. During tetanic activation of muscle there is a decline in quantal content/number, which can be a challenge to effective neuromuscular transmission^28,29^. Interestingly, blood concentrations of AEA increase after athletes exercise^30,31^. It is conceivable that during sustained exercise, muscles produce AEA and that this serves to raise quantal size and thereby compensate for the natural decline in quantal content, as a physiological response. Our study reports a cannabinoid-induced increase in the content of ACh within each vesicle rather than change in the number of vesicles released per nerve impulse. The distinct mechanism through which vesicle filling is regulated, independently of the control of quantal release, will require further detailed investigation.

The pharmacological profile of the cannabinoid-induced effects suggests that WIN activates different pathways at the mammalian NMJ, compared to its known effects in the CNS. In the presence of the CB1 inverse agonist AM251, WIN produced no significant increase in quantal amplitude. This was also the case for the CB2 inverse agonist AM630 but AM630, on its own, significantly raised the mEPP amplitude. Our preliminary pharmacological investigations, testing the effects of AM630 and AM251 on mEPP amplitudes, will require further careful pharmacological assessment. CB1 ligands are known to behave either as agonists or antagonists depending upon the interaction of the receptor with specific Gi subunits. For example, (R)-methanandamide behaves as an inverse agonist for CB1-Gi1 and -Gi2, but as an agonist for CB1-Gi3 complexes^32^. Moreover, in the pineal gland, nucleus accumbens and globus pallidus CB1 and CB2 receptors can form heteromers with bidirectional cross-antagonism of downstream signalling pathways and activation of either CB receptor can lead to modulation of the partner receptor^33^. The pathways typically engaged by CB receptors include regulation of cAMP levels, stimulation of MAPK activity, elevation of intracellular calcium and opening of K^+^ channels^6^. A recent study reported that the neuropeptide CGRP can increase mEPP amplitudes at the mouse NMJ via a PKA-inhibitor (H-89)-mediated process. This CGRP effect was blocked by vesamicol^34^ suggesting that it too was dependent upon synaptic vesicle refilling. Given the emerging complexity of cannabinoid signal transduction, further detailed pharmacological studies will be needed to: (i) characterise the types of cannabinoids that elevate quantal size, (ii) identify the receptor(s) involved, and (iii) characterize the second messenger pathways responsible for cannabinoid-induced increase in quantal size at the mammalian NMJ.

Cannabinoid drugs have already been approved for the treatment of nausea in chemotherapy patients and clinical trials are underway to test for treatment of spasticity and other muscle-debilitating symptoms of multiple sclerosis^35–37^. The increasing usage of cannabinoids in a variety of medical conditions^38,39^ highlights the crucial need to better understand the physiological roles of cannabinoids in the periphery. Here we reveal evidence of their involvement in regulating neuromuscular transmission, and a possible therapeutic potential for cannabinoid signaling in myasthenia gravis.

## Methods

### Animals and Ethical approvals

Female C57BL/6 J mice (6–10 weeks) obtained from (Animal Resources Centre, Murdoch, WA Australia) were used in this study. Mice were killed with an overdose of pentobarbitone (30 mg intraperitoneal; CenVet Australia). All mouse experiments described in this paper were conducted with the approval of The University of Sydney Animal Ethics Committee (approval number K22/10-2011/3/5619) in compliance with the NSW Animal Research Act 1985 and the Australian Code of Practice for the Care and Use of Animals for Scientific Purposes 7th Edition NH&MRC 2004. In relation to the collection of plasma, informed, written consent was obtained from patients in accordance with the Declaration of Helsinki (5th revision, 2004). The project was approved by the Human Research Ethics Committee of the Sydney South West Area Health Service.

### Endplate potential recordings

The diaphragm with the phrenic nerve attached was quickly dissected, pinned on a Sylgard-coated dish and bathed in oxygenated Ringer’s solution containing physiological calcium levels (in mM: NaCl-136, KCl-5, NaH_2_PO_4_-1, NaHCO_3_-12.8, MgCl_2_-1, CaCl_2_ and glucose-10, pH adjusted to 7.3). Spontaneous miniature endplate potential (mEPP) and nerve-evoked endplate potential (EPP) recordings were made at room temperature as previously described^28,40^. Contraction was blocked using the muscle sodium channel blocker, µ-conotoxin GIIIb (1 μM μCTX, Peptide Institute, Japan). EPP and mEPP recordings were started 1–2 hrs after the diaphragm was placed in the bath solution. Spontaneous mEPP amplitudes were recorded for 1–3 min and were normalized to a resting potential of −80 mV. A train of 40 stimuli (1 Hz) was used for EPP analyses (average amplitude; 20 stimuli for the vesamicol and bafilomycin experiments). EPP amplitudes were normalized to −80 mV and then corrected for non-linear summation^41^. Quantal content was calculated by dividing the normalized and corrected EPP amplitudes by the normalized mEPP amplitude for each muscle fiber. Recordings were made from 12–18 muscle fibers from each muscle to derive mean mEPP and EPP values for each sample. Only fibers with a stable resting membrane potential (RMP) ≤ −60 mV were analyzed. To calculate the 90–10% decay times before and after pyridostigmine treatment the average decay time for all sample traces (irrespective of peak amplitude) for n = 3–4 mice was compared.

### Drug treatments

In all experiments, drug stock solutions were diluted into the physiological saline solution bathing the preparation. Drugs were obtained from Sigma (MO, USA). Stock solutions for R(+)-WIN 55,212 (WIN), AM251, AM630, L-+/-vesamicol, bafilomycin, anandamide and URB597 were first dissolved in DMSO, stored at −20 °C, and added to the bath 1–2 hrs before the start of recordings. This drug exposure time allowed for dissection of the tissue and set up of the *ex-vivo* preparation prior to start our recordings. It also ensured enough time for the drugs in the bath to diffuse through the *ex-vivo* diaphragm muscle preparations. Total DMSO in the Ringers solution was maintained at <0.01%. Control preparations received the same concentration of DMSO vehicle. To test the effect on presynaptic vesicle filling, control recordings were obtained first and then L-+/-vesamicol or bafilomycin were added to the bath. After incubation for approximately 30 min the cannabinoid agonist WIN was also added to the bath before recordings were recommenced a further 60–90 min later.

For the *in vivo* experiments, compound muscle action potentials (CMAP) were recorded from myasthenic mice (see below). The mice then received an intraperitoneal injection of R(+)-WIN 55,212 (5 mg/kg; diluted in 0.9% saline) and a second set of CMAP recordings were made 3 hrs later. At the dose employed mice showed some signs of hyperactivity but did not display behavior suggestive of intoxication (such as nasal secretions or muscle fasciculation). After the phrenic nerve-diaphragm preparation was dissected it was thoroughly washed for 1.5 hrs in Ringer’s solution to remove traces of the injected WIN prior to baseline EPP recordings. The acute effects of WIN were then tested by adding WIN to the bath solution for 1.5 hrs.

### Confocal microscopy, NMJ morphometry and FRET

Immunostaining, imaging (Zeiss LSM510 Meta confocal microscope) and endplate morphometry was performed as described previously^40,42^. For comparison of AChR staining intensities, sections were processed together and examined during the same imaging acquisition using identical imaging settings. Confocal Fluorescence Resonance Energy Transfer (FRET) was used to compare the extent to which AChRs were tightly packed within endplate AChR clusters (<10 nm AChR-AChR spacing) as described previously^43^. FRET efficiency was calculated from the increase of the fluorescence intensity of the donor after the acceptor fluorophor was selectively photobleached.

### Electron microscopy and synaptic vesicle measurements

Diaphragms prepared as described above were divided into two hemidiaphragms. The right hemidiaphragm was bathed in WIN (10 µM) for ~2 hrs while the left hemidiaphragm was incubated in control solution (without WIN). Samples were then immersion fixed in 4% paraformaldehyde +2.5% glutaraldehyde in 0.05 M cacodylate buffer (containing 2% sucrose and 0.15 mM CaCl2, pH 7.4) for 10 min (modified from)^44^. Diaphragms were then sliced into approx. 1–2 mm strips (perpendicular to the main nerve) and, fixed for a further 45 min followed by heavy metal staining^45^. Finally, each strip of diaphragm was flat embedded in EPON resin and polymerized overnight at 60 °C. An ultramicrotome (Ultracut7, Leica, Germany) was used to produce sections for LM and EM observations. Semi-thin sections (500 nm) across the entire length of the diaphragm were produced, stained with Toluidine blue and observed under a light microscope: this step aided in trimming the diaphragm down to only the areas located within 2–3 mm of the main nerve (where most NMJs are located). Ultra-thin sections (70 nm) were then produced from the trimmed area, collected on 200 mesh copper grids, stained with Uranyl acetate and Lead citrate for 10 min each before being observed with a TEM, operating at 120 kV (JEM 1400, JEOL, Japan).

Measurements of vesicle diameter were performed on digital micrographs by an operator who was blind to the treatment group and following the protocol outlined in Karunanithi *et al*.^26^. Briefly, the outer diameters of synaptic vesicles were measured for at least 200 vesicle profiles, which was sufficient to obtain a characteristic distribution^46^. Only clearly distinguished vesicles that were circular in shape, had a grey core and a uniform membrane were used for the analysis. At least 15 NMJs were sampled from each of four hemidiaphragms/mice. A greater number of micrograph images were analysed from WIN-treated muscles than from control muscles (Control: n = 597 vesicles; WIN: n = 2098 vesicles). Vesicle diameters were corrected using the method of Froesch so as to take into consideration EM section sampling^26,47^. Volumes were calculated assuming spherical geometry (Volume = 4/3*π*radius^3^) based upon the inner diameter of the vesicles (outer diameter minus twice the vesicle membrane thickness (8.9 ± 0.19 nm; n = 103 vesicles).

### Mouse model of anti-MuSK myasthenia gravis

The passive IgG transfer model of anti-MuSK myasthenia gravis was described previously^40,48,49^. Briefly, mice received daily injections of IgG from anti-MuSK-positive patient 4 (45 mg/day i.p., patient suffering grade 4B weakness - Myasthenia Gravis Foundation of America; IgG batch AM4.4). Passive transfer of IgG from this patient was previously reported to cause overt myasthenic weakness after 12 daily injections^50^.

### Electromyography

Compound muscle action potentials (CMAP) were recorded from the gastrocnemius muscle during repetitive stimulation of the sciatic nerve, as previously described^40^. Briefly, the mouse was first anesthetized with 2–3% isoflurane/oxygen. Two ~3 mm custom-made single monopolar recording electrodes were glued to the surface of the skin: one over the dorsal aspect of the gastrocnemius muscle and the second electrode at the ankle of the same hind limb. Electrolyte gel (VIASYS Healthcare, Madison, USA) was applied directly at the electrode sites. Stimulation of the sciatic nerve was accomplished via a 4 mm incision in the sciatic notch and by placing the nerve on custom-made silver hook electrodes (0.6 mm diameter). Ten stimuli were delivered at 3 impulses/sec and three such trains recorded from each muscle were averaged.

### Statistics

Graphpad Prism (GraphPad Software, CA, USA) was used for statistics and to visualize the differences between the treatment groups. EPP recordings before and after drug treatment were made from the same muscle and differences between the means were evaluated using a paired Student’s t-test. For all statistical tests significance was taken as P < 0.05. Unless otherwise indicated the data was symmetrically distributed with equivalent variances and values are presented as the mean ± standard error of the mean (SEM).

## Electronic supplementary material

Supplementary file
