# Supplementary material for: Cannabinoid-induced increase of quantal size and enhanced neuromuscular transmission
Source: Sci Rep. 2018 Mar 16;8:4685. doi: 10.1038/s41598-018-22888-4 (PMC5856814; doi:10.1038/s41598-018-22888-4)
Supplement: Supplementary file 1 — Supplementary file [file 41598_2018_22888_MOESM1_ESM.docx]

**Cannabinoid-induced increase of quantal size and enhanced neuromuscular transmission**

Marco Morsch^1,2*^, Dario A. Protti^2^, Delfine Cheng^3^, Filip Braet^3,4^, Roger Chung^1^, Stephen W. Reddel^5^, William D. Phillips^2*^

^1^ Department of Biomedical Sciences, Faculty of Medicine & Health Sciences, Macquarie University, Sydney, NSW, 2109, Australia.

^2^ Discipline of Physiology and Bosch Institute, The University of Sydney, NSW, 2006, Australia.

^3^ School of Medical Sciences (Discipline of Anatomy and Histology), The Bosch Institute, The University of Sydney, NSW, 2006, Australia.

^4^ Australian Centre for Microscopy & Microanalysis (ACMM), The University of Sydney, NSW, 2006, Australia.

^5^ Departments of Molecular Medicine & Neurology, Concord Clinical School, The University of Sydney, NSW, 2006, Australia.

**Morsch et al. Supplementary material**

**
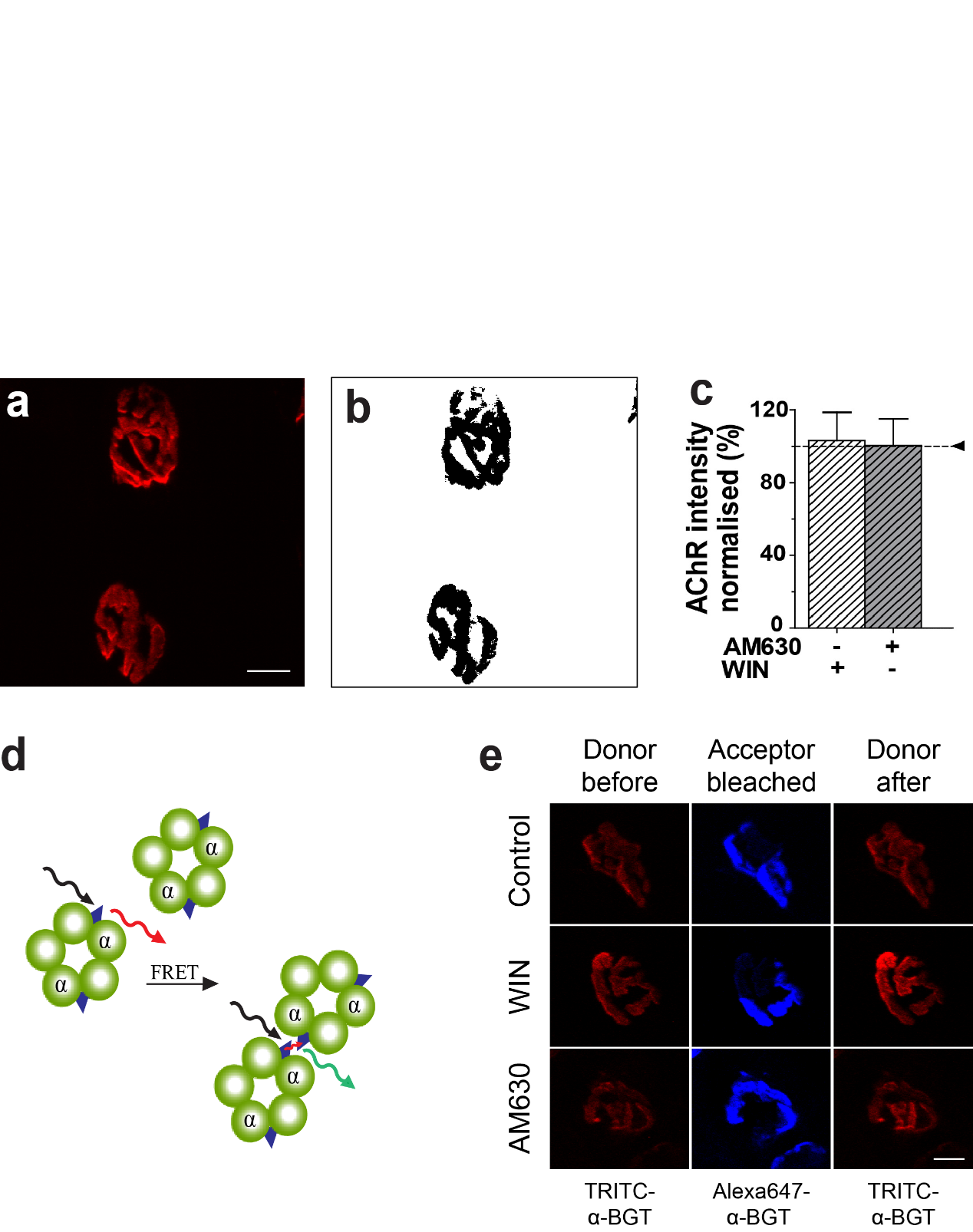
**

**Supplementary Fig. S1:** **Acetylcholine receptor density did not change with cannabinoid treatment**

**(a)** Representative enface image of postsynaptic AChR at motor endplates. Longitudinal sections of the diaphragm muscle were labeled with Alexa-555-α-bungarotoxin. Confocal microscope image stacks of endplates were collected and projected to two-dimensional maximum projection images. **(b)** Binary image generated by thresholding the image in panel a, illustrating how area was measured. **(c)** Average intensity of fluorescent labeling for AChR at endplates sampled from muscles treated with either AM630 or WIN compared to untreated control endplates. Fluorescence intensity is expressed as a percentage of untreated control endplates (horizontal line). **(d)** Principle of our FRET approach to quantify postsynaptic receptor density. In this assay, efficient FRET is detected when adjacent pentameric AChRs are closely packed in the postsynaptic membrane. **(e)** Representative FRET examples for the treatment with WIN and AM630 (CB_2_ anta). AChRs were labeled with a mixture of TRITC-α-BGT (red) and Alexa647-α-BGT (blue). The photobleaching of acceptor method was used to assess FRET efficiency and was calculated from the increase of the fluorescence intensity of the donor after the acceptor fluorophor was selectively photobleached^21^.

**
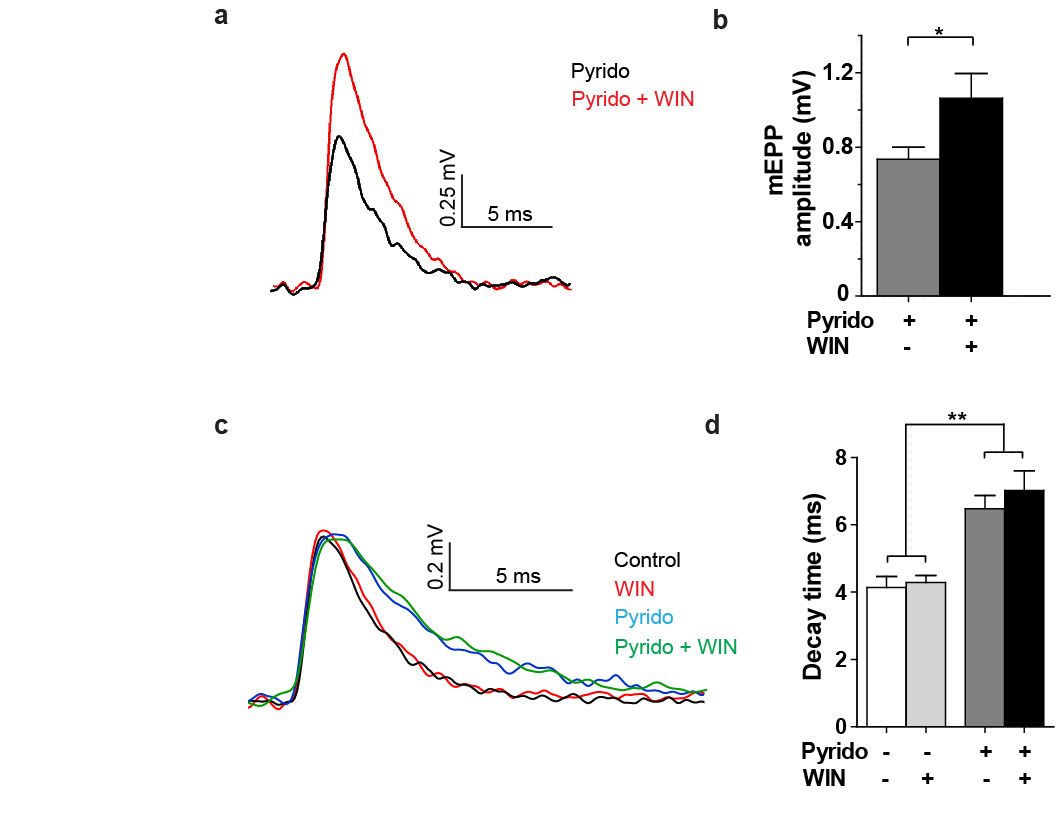
**

**Supplementary Fig. S2:** **WIN does not increase mEPP amplitude by inhibiting acetylcholinesterase**.

**(a)** Representative mEPP traces illustrating the increase in amplitude after WIN treatment in the presence of 10µM pyridostigmine (black trace pyridostigmine; red trace pyridostigmine + 10 µM WIN). **(b)** Mean mEPP amplitude analysis. The presence of 10 µM pyridostigmine did not block a WIN-induced 1.4-fold increase in the average mEPP amplitude (*P<0.05). **(c)** Examples of mEPP traces recorded from fibers in an untreated diaphragm muscle (black trace) and after exposure of the muscle to bath-applied 10 µM WIN (red trace), 10 µM pyridostigmine (blue trace), or pyridostigmine plus WIN (green trace). For the purpose of illustrating the prolonged mEPP decay that was consistently observed in the presence of pyridostigmine, traces of similar amplitude were superimposed. **(d)** Mean decay time (90-10%) for all sampled traces (irrespective of amplitude) was significantly prolonged by pyridostigmine while WIN had no significant effect upon decay time. Bar graphs represent the mean ± SEM for n=3-4 mice and measurements of 8 to 17 fibers for each mouse (*P<0.05; paired Student's t-test in B and one-way ANOVA in C).

**
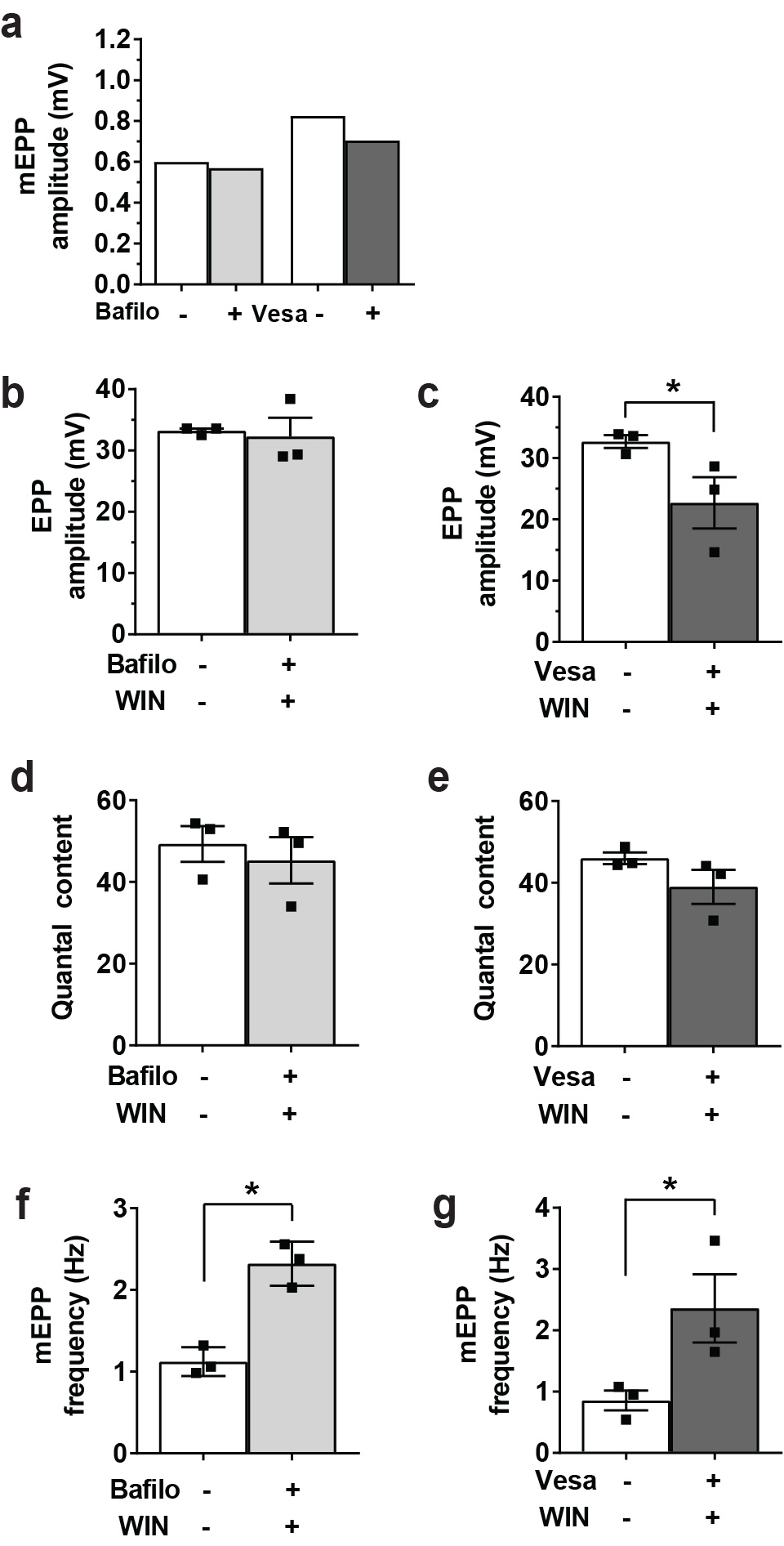
**

**Supplementary Fig. S3:** **Effects of blockers of presynaptic vesicle filling upon synaptic potentials**

Paired bars show the effect of the vesicular proton pump inhibitor bafilomycin (0.1 µM) and the vesicular ACh transporter inhibitor vesamicol (4 µM), compared to untreated controls. **(a)** As previously reported neither bafilomycin nor L-+/--vesamicol had any significant effect on mEPP amplitude when applied on their own (n=11-14 fibers from 1 mouse). **(b)** In the presence of bafilomycin WIN produced no change in EPP amplitude while L-+/--vesamicol produced a decrease in EPP amplitude **(b). (d-e)** Quantal content was not altered by the combination of WIN 55,212 (10 µM) plus bafilomycin (0.1 µM) or WIN (10 µM) plus vesamicol (4 µM). **(f-g)** The frequency of spontaneous mEPPs was significantly increased by the combination of WIN plus Bafilomycin and WIN plus L-+/--vesamicol. In b-g each bar represents the mean ± SEM with measurements n=3 muscles with 12 to 16 fibers sampled from each muscle (*P<0.05; **P<0.01; ***P<0.001; paired Student's t-test).

**Supplementary Table 1:** **Quantitative data analysis on vesicle diameters and volumes as determined by electron microscopy.**

**Supplementary Table S2: Effects of WIN, AN251 and AM630 on synaptic potentials**

|  | | |  |  |  |  |  |  |  |  |
| --- | --- | --- | --- | --- | --- | --- | --- | --- | --- | --- |
|  | Treatment | | | | | | | | | |
| **mEPPs** | **Naive control** | **WIN** |  | **Naive control** | **AM251** | **AM251 + WIN** |  | **Naive control** | **AM630** | **AM630 + WIN** |
| Number of fibres recorded (mice) | 140 (10) | 142 (10) |  | 44 (4) | 46 (4) | 50 (4) |  | 30 (3) | 30 (3) | 30 (3) |
| Frequency (Hz) | 1.336 | 1.627 |  | 1.177 | 0.9844 | 0.9313 |  | 0.8638 | 0.8751 | 1.094 |
| Resting Membrane Potential (mV) | -65.97 | -65.31 |  | -65.48 | -65.2 | -64.72 |  | -64.27 | -65.23 | -66.63 |
| mEPP rise time (ms) | 1.118 | 1.099 |  | 0.9166 | 0.9528 | 1.133 |  | 0.8913 | 1.17 | 0.8725 |
| mEPP fall time (ms) | 4.067 | 4.117 |  | 3.795 | 3.745 | 4.032 |  | 3.31 | 3.559 | 3.36 |
|  |  |  |  |  |  |  |  |  |  |  |
|  |  |  |  |  |  |  |  |  |  |  |
| **EPPs** | **Naive control** | **WIN** |  |  |  |  |  |  |  |  |
| Number of fibres recorded (mice) | 65 (5) | 76 (5) |  |  |  |  |  |  |  |  |
| Resting Membrane Potential (mV) | -66.34 | -64.02 |  |  |  |  |  |  |  |  |
| EPP rise time (ms) | 0.8517 | 0.8969 |  |  |  |  |  |  |  |  |
| EPP fall time (ms) | 5.051 | 4.744 |  |  |  |  |  |  |  |  |
